# Cobalamin production by *Lactobacillus coryniformis*: biochemical identification of the synthetized corrinoid and genomic analysis of the biosynthetic cluster

**DOI:** 10.1186/s12866-016-0854-9

**Published:** 2016-10-13

**Authors:** Andrea Carolina Torres, Verónica Vannini, Julieta Bonacina, Graciela Font, Lucila Saavedra, María Pía Taranto

**Affiliations:** Centro de Referencia para Lactobacilos (CERELA)-CONICET, San Miguel de Tucumán, Tucumán Argentina

**Keywords:** *Lactobacillus*, Cobalamin production, Coenzyme B_12_ gene cluster organization

## Abstract

**Background:**

Despite the fact that most vitamins are present in a variety of foods, malnutrition, unbalanced diets or insufficient intake of foods are still the cause of vitamin deficiencies in humans in some countries. Vitamin B_12_ (Cobalamin) is a complex compound that is only naturally produced by bacteria and archea. It has been reported that certain strains belonging to lactic acid bacteria group are capable of synthesized water-soluble vitamins such as those included in the B-group, as vitamin B_12_. In this context, the goal of the present paper was to evaluate and characterize the production of vitamin B_12_ in *Lactobacillus coryniformis* CRL 1001, a heterofermentative strain isolated from silage.

**Results:**

Cell extract of *L. coryniformis* CRL 1001, isolated from silage, is able to correct the coenzyme B_12_ requirement of *Salmonella enterica* serovar Typhimurium AR 2680 in minimal medium. The chemical characterization of the corrinoid-like molecule isolated from CRL 1001 cell extract using HPLC and mass spectrometry is reported. The majority of the corrinoid produced by this strain has adenine like Coα-ligand instead 5,6-dimethylbenzimidazole. Genomic studies revealed the presence of the complete machinery of the anaerobic biosynthesis pathway of coenzyme B_12_. The detected genes encode all proteins for the corrin ring biosynthesis and for the binding of upper (β) and lower (α) ligands in one continuous stretch of the chromosome.

**Conclusions:**

The results here described show for the first time that *L. coryniformis* subsp. *coryniformis* CRL 1001 is able to produce pseudocobalamin containing adenine instead of 5,6-dimethlbenzimidazole in the Coα-ligand. Genomic analysis allowed the identification and characterization of the complete *de novo* biosynthetic pathway of the corrinoid produced by the CRL 1001 strain.

## Background

Lactic acid bacteria (LAB) are a heterogeneous group of bacteria extensively used as starter cultures for the fermented foods development. Because of their metabolism, this group of bacteria can improve the safety, shelf life, nutritional value, flavour and overall quality of fermented products. Some strains have shown to exert a large range of beneficial properties in humans, and they are frequently used as probiotic microorganisms. In addition, certain LAB strains are able to produce and release compounds with biological activity in foods, sometimes referred as nutraceuticals [1]. These micronutrients are used as a cofactor in numerous enzymatic reactions, as it is the case of vitamin B_12_.

Conceptually, the definition of vitamin B_12_ generally describes a type of cobalt corrinoid, belonging to the cobalamin (Cbl) group. On the other hand, vitamin B_12_ is the form of the vitamin obtained during process of industrial production but this form not exists in the nature [2]. Naturally, this compound is found as desoxyadenosilcobalamin (coenzyme B_12_), methylcobalamin or pseudocobalamin, among other forms. Concerning the structure, the cobalamin molecule present 3 parts main: (i) the central corrinic ring with the four ligands of a cobalt ion, (ii) a superior (or beta) ligand that is attached to adenosyl o methyl group, and (iii) the lower ligand (or alfa), usually dimethylbenzimidazole (DMB). It has been described that in some anaerobic bacteria, the adenine and other ligands can replace DMB giving as a result pseudocobalamin (pseudo-B_12_) and other active cofactors [3]. This soluble compound is one of the most complex non-polymeric macromolecules produced in the cell and only a reduced group of bacteria and archaea are able to synthesize it. The bacteria present in the rumen of cattle, sheep and other ruminants are the responsible of its synthesis. Humans do not have such microbiota in their large intestine and must absorb the coenzyme from natural sources such as meat, fish, eggs, and pharmaceutical products [4]. A diet sufficient in vitamin B_12_ is essential to prevent severe pathologies (megaloblastic anaemia, pancytopenia, peripheral neuropathy, increased risk of myocardial infarction and stroke, others) some of which are irreversible [5–8]. Thus, the use of vitamin-producer bacteria appears as an appropriate alternative to produce innovative foods with added nutritional value [9]. However, the production of vitamin B_12_ is strain specific and an unusual trait present in certain strains of the *Lactobacillus* genus. The production of this metabolite by *Lactobacillus reuteri* strains has been described for the first time by us and confirmed later by others authors [10–13]. Moreover, we validated that the corrinoid (pseudo-B_12_) produced by *L. reuteri* CRL 1098 is effective in preventing the hematological and neurological pathologies (anaemia, growth retardation, others) caused by a diet deficiency in vitamin B_12_ in a combined animal model (pregnant mice and their offspring) [14, 15]. More recently, De Angelis et al. [16] described the production and the genetic organization of the complete *de novo* biosynthetic pathway of vitamin B_12_ in *Lactobacillus rossiae* DSM 15814. Among other lactobacilli, previous evidence suggested that two strains of *L. coryniformis* (CECT 5711 and strain 394) are capable of producing the antimicrobial compound reuterin [17, 18], an enzymatic pathway that involves a cobalamin-dependent enzyme, the glycerol dehydratase [19]. No further analysis related to vitamin B_12_ production was carried out in those studies. Based on this previous evidence, the purpose of the present study was to evaluate the vitamin B_12_ production in *L. coryniformis* CRL 1001, a heterofermentative strain isolated from silage. Firstly, the compound with cobalamin activity was isolated and biochemically characterized. Next, the genome of the CRL 1001 strain was sequenced by whole genome shotgun (WGS) sequencing strategy and the identification of putative biosynthetic operon involved in the synthesis of the corrinoid was performed by *in silico* analysis. Finally, a detailed comparative study of the cobalamin gene cluster of *L. coryniformis* CRL 1001 with those of previously known vitamin-producer strains was performed. The results of this research provide the first evidence of vitamin B_12_ synthesis by a *L. coryniformis* strain.

## Results and discussion

### Cobalamin production by *L. coryniformis* CRL 1001

In order to evidence the production of cobalamin by *L. coryniformis* CRL 1001, we use *S.* Typhimurium AR 2680 (*metE cbiB*) [20] as indicator strain, grown in minimal medium without vitamin B_12_ according the methodology described by Taranto et al. [12]. The results of the bioassay showed that the cobalamin requirements of the indicator strain were corrected when Cell Extracts (CE) of *L. coryniformis* CRL 1001 were added; the halo diameter obtained was similar to that obtained with a solution of standard cyanocobalamin (CN-Cbl). On the other hand, no growth was detected when the CE from *Lactobacillus plantarum* ATCC 8014 (negative control strain) was used. These results would indicate that *L. coryniformis* CRL 1001 is able to produce compounds with cobalamin activity.

**Fig. 1:**
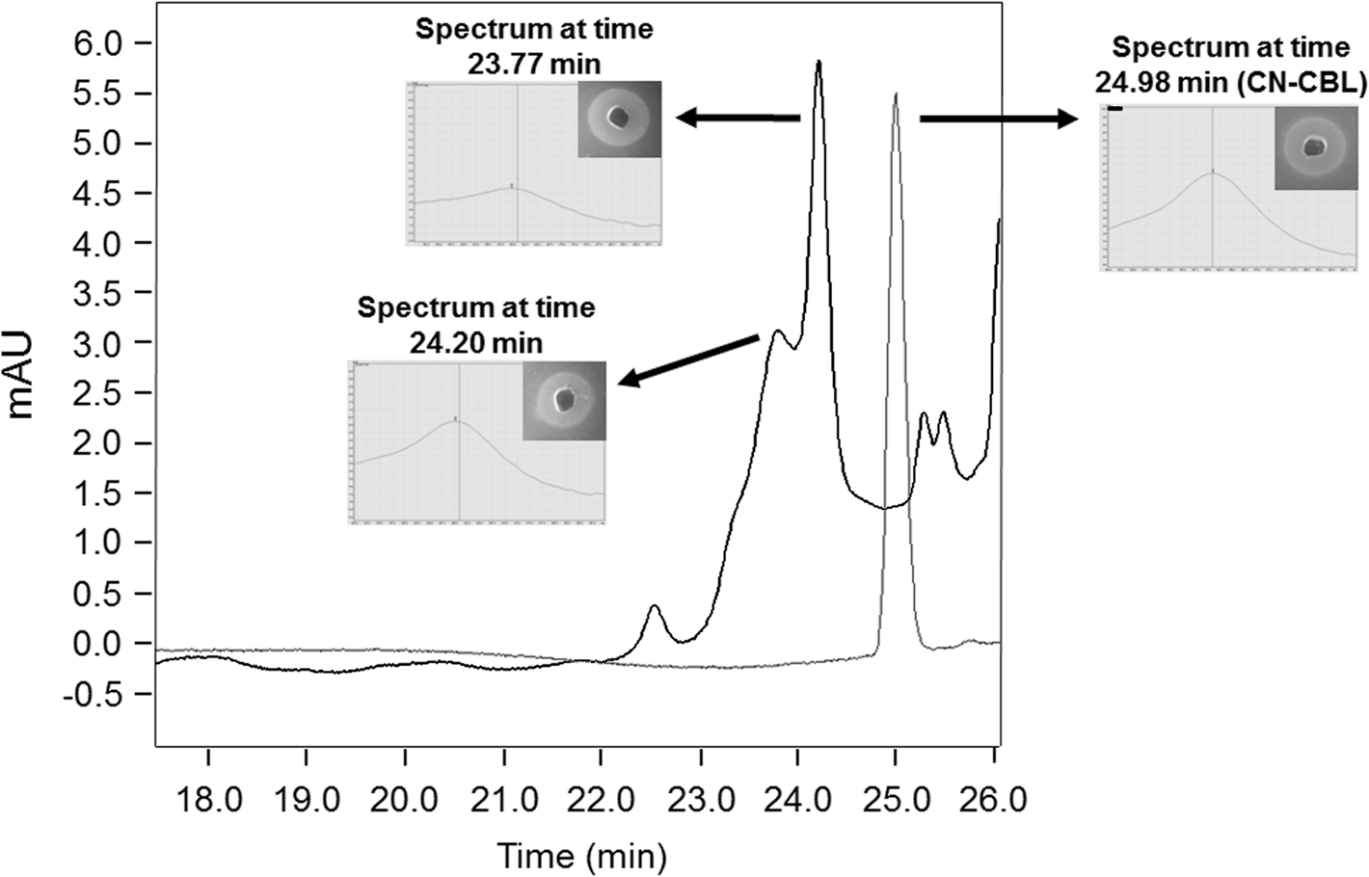
RP-HPLC chromatogram of the cell extract (CE) from *L. coryniformis* CRL 1001 (black); CN-Cbl standard (grey). UV-DAD spectrum and bioassay images (annex inset) of CN-Cbl standard and each corrinoid produced by CRL 1001 strain

To identify the compound with cobalamin activity produced by *L. coryniformis* CRL 1001, the CE was analysed by RP-HPLC. The UV-DAD spectra data revealed the presence of two main peaks that showed similar characteristics with the spectrum obtained for the peak belonging to the CN-Cbl standard. The Retention Times (RT) of these peaks were slightly lower (23.77 min and 24.20 min) to the RT of standard solution of CN-Cbl (24.98 min) (Fig. 1). However, when these peaks were collected and analysed using the bioassay, they showed the same ability of vitamin B_12_ complementation than the CN-Cbl standard. For this reason, we continued with the identification of both peaks.

### Characterization of the corrinoid produced by *L. coryniformis* CRL 1001

Corrinoid compounds separated by HPLC were analysed by Liquid chromatography–electrospray ionization/tandem mass spectrometry (LC/ESI–MS/MS). The mass module parameters were fine-tuned and optimized with commercial standard of CN-Cbl. The transition 678.3 (m/z) [M + 2H^+^]^++^ to 358.7 (m/z) corresponds to the lower ligand, whose DMB is the aglycon attached to ribofuranose 3-phosphate whereas the transition 678.3 (m/z) [M + 2H^+^]^++^ to 146.9 (m/z) corresponds to the base DMB. Data transition for pseudo-B_12_ were estimated and calculated according the bibliography as follows: 672.5 (m/z) [M + 2H^+^]^++^ to 347.8 (m/z); this transition corresponds to the lower ligand where adenine is the aglycon attached to ribofuranose 3-phosphate, and the transition 672.5 (m/z) [M + 2H^+^]^++^ to 135.9 (m/z) corresponds to the base adenine [21]. Both transitions were sought in the MS and MS/MS spectra of the peaks with cobalamin activity.

**Fig. 2:**
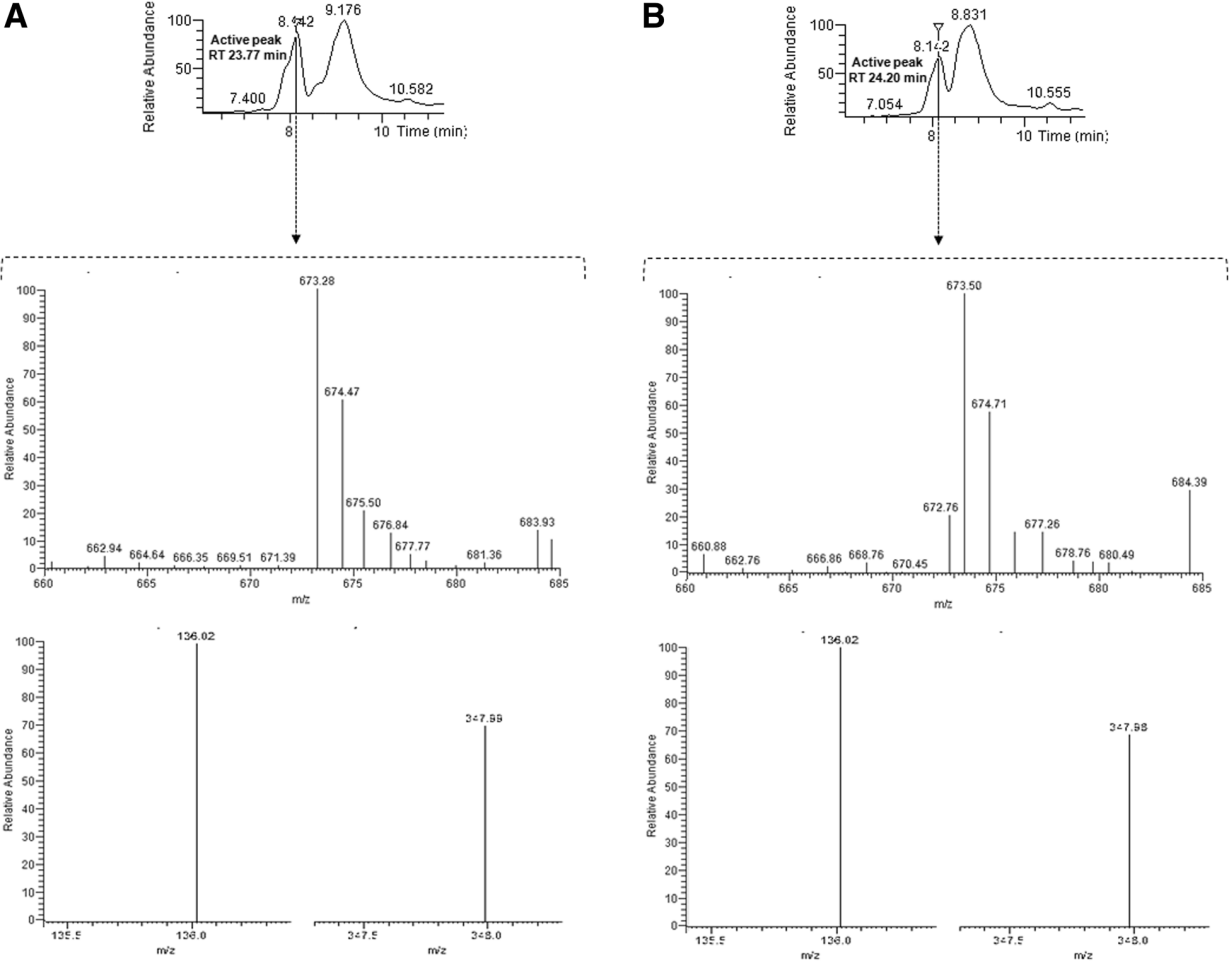
Liquid chromatography–electrospray ionization/tandem mass spectrometry (LC/ESI–MS/MS) chromatograms of peaks with cobalamin activity from *L. coryniformis* CRL 1001. The transitions are shown together (SRMs, 672.5 m/z -- > 136.0 m/z calculated for adenine y 672.5 m/z -- > 348.0 m/z correspond to the lower ligand in which adenine is the aglycon attached to ribofuranose 3-phosphate). Total ion chromatogram (TIC) of the two active peaks, RT 23.77 min (**a**) and RT 24.20 min (**b**) and MS/MS spectrum from m/z 660 to 685 of the peak with RT 8.142 min with MS/MS spectrum showing the transitions

The MS spectra of the active peaks obtained by HPLC indicated that a double charged ion with an approximately m/z of 673 [M + 2H^+^]^++^ was prominent in the two spectra (Fig. 2). The MS/MS spectrum indicated that the dominant ions with a value approximate m/z 347.8 were attributable to the substitution in the lower ligand where the adenine is the aglycon attached to ribofuranose 3-phosphate.

The MS showed that the molecular species (corrinoid) synthesized by *L. coryniformis* CRL 1001 corresponds mainly to Coα-[α-(7-adenyl)]-Coβ-cyanocobamide, commonly known as pseudo-B_12_, an analogue with a structure very similar to vitamin B_12_. In this corrinoid, the DMB moiety is substituted by adenine as lower ligand. This compound is a physiologically important form of the vitamin B_12_ in various microorganisms [22].

The concentration of pseudo-B_12_ produced by *L. coryniformis* CRL 1001 was calculated indirectly using a standard curve made with different concentrations of a commercial CN-Cbl. The area of the peaks obtained in the chromatograms corresponds to approximately 0.94 μg mL^−1^ of pseudo-B_12_ produced.

### Genome identification of vitamin B_12_ cluster in *L. coryniformis* CRL 1001

The genome sequence of *L. coryniformis* CRL 1001 consisted on 133 contigs with a total size of 2,829,178 base pairs and an average GC content of 42 %. The structural and functional annotation performed with the Rapid Annotations using Subsystems Technology (RAST) server [23] allowed us to identify 3341 coding sequences (CDS) and 82 structural RNAs (58 tRNAs) [23]. Additionally, it was observed that 332 subsystems that include the 44 % of the identified CDS are represented in the chromosome.

**Fig. 3:**
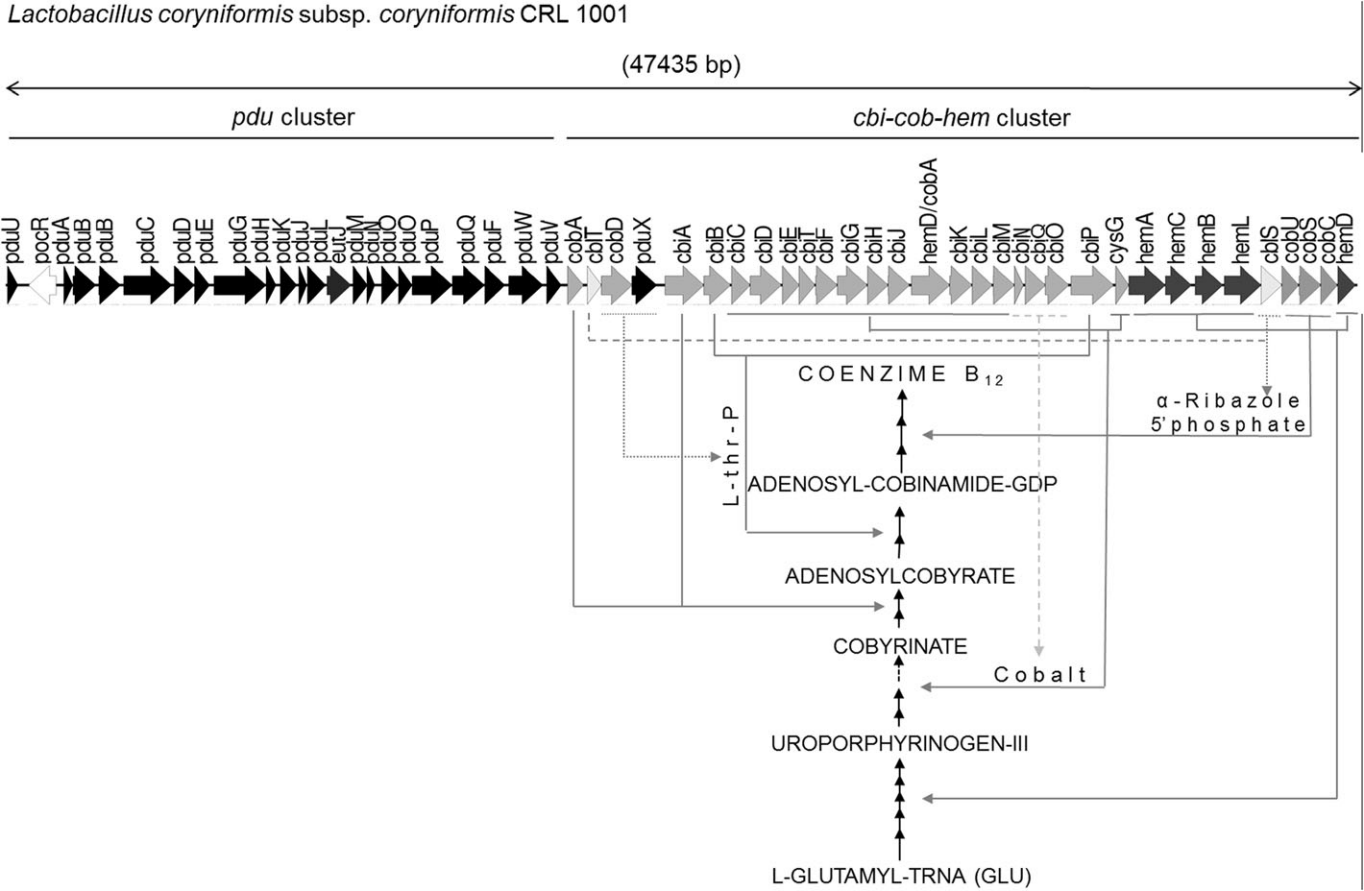
Schematic representation of the *pdu* and *cbi-cob-hem* clusters of *L. coryniformis* CRL 1001. The arrows represent genes involved in: synthesis of uroporphyrinogen III (dark grey), synthesis of cobinamide, lower ligand and cobalt transport (light grey), propanediol degradation (black) and *pocR*, a transcriptional regulator (white). On the simplified B_12_ synthesis pathway scheme, genes encoding proteins involved in synthesis of coenzyme (full lines) and synthesis and transport of intermediaries (incomplete lines) are highlighted

*In silico* genomic analyses revealed the presence of 32 open reading frames (ORFs) related to the coenzyme B_12_ production (*cbi*, *cob*, *hem* and *cbl* gene cluster) located immediately adjacent to the *pdu* operon (22 ORFs) which encodes the enzymes necessary for the use of glycerol or propanediol as carbon source (Fig. 3). As described in other species, the close association of these two gene clusters is probably a reflection of the requirements of cobalamin or its corrinoid derivatives for the use of propanodiol [24]. Almost all predicted genes (*pdu*, *cbi*, *cob* and *hem*) are in the same orientation, except for *pocR*, which encodes for a transcriptional regulator with 45 % identity and 69 % positive matches at the amino acid level, with its homolog in *L. reuteri* JCM 1112 [25]. Interestingly, the two clusters (*pdu* and *cbi*-*cob*-*hem*) have a few intergenic regions and their average G + C content (52 %) differ from that of the draft genome sequence of *L. coryniformis* CRL 1001 (42 %). As described by Morita et al. [10] for *L. reuteri* JCM 1112, this data could explain the existence of a genomic island acquired through horizontal gene transfer.

Among *pdu* genes, *pduC, pduD* and *pduE* were found. These genes encode for the three subunits of the glycerol dehydratase, an enzyme that requires cobalamin as a cofactor for performing it catalytic activity [19]. Additionally, a gene coding for the L-threonine kinase PduX, was localized. This protein, described in *Salmonella enterica*, transfers a phosphoryl group to a free L-threonine and it is involved in *de novo* synthesis of Adenosyl cobalamin and the assimilation of cobyric acid (Cby) [26].

Downstream of the *pdu* operon, seventeen *cbi* genes (*cbiA, B, C, D, E, T, F, G, H, J, K, L, M, N, Q, O,* and *P*) and *cysG* gene were *in silico* identified*.* The proteins encoded by these genes are required in most of the reactions that take place in the cell during the corrinoid ring synthesis of the cobalamin molecule [27].

Regarding *cob* genes *cobA*, *D, U, S* and *C* were found. *cobA* encodes an adenosyltransferase, a binding protein of adenosyl group as β-ligand [28]. The gene *cobD* encodes an L-threonine-O-3-phosphate descarboxylase that is involved in the amino-propanol arm synthesis [29] while *cob U*, *S* and *C* encode the proteins that participate in the attachment of the amino-propanol arm and in the assembly of the nucleotide loop that connects the lower cobalt ligand to the corrinoid ring [30].

Finally, downstream of the *cbi* and *cob* genes, five *hem* genes (*hem A*, *C*, *B*, *L* and *D*) were detected in a similar way as observed for other microorganisms that produce cobalamin anaerobically [31]. The proteins encoded by these genes are involved in the first steps of the cobalamin biosynthesis, the synthesis of uroporphyrinogen III from L-glutamyl-tRNA(glu). At the same time, the *hemD/cobA* gene, encoding a single polypeptide with both uroporphyrinogen III synthase and methyltransferase activity was detected [32].

Interestingly, the genome of *L. coryniformis* CRL 1001 lacks *cobT* gene, contrary to that observed in the cobalamin biosynthetic cluster of *L. reuteri* CRL 1098 [11]. Instead, two *cbl* genes were detected*, cblS* and *cblT*, encoding for a α-ribazole phosphoribosyl kinase and a α-ribazole transporter, respectively. These results might suggest an alternative pathway for α-ribazole salvaging and the α-ribazole-P synthesis in *L. coryniformis* CRL 1001, as it is described in *Listeria* [33].

### *In silico* comparative analysis of the biosynthetic cluster of vitamin B_12_

The cobalamin biosynthetic gene cluster has been identified in a few *Lactobacillus* species, including *L. reuteri* [11] and more recently in *L. rossiae* [16]. Both strains are obligate hetero-fermentative LAB [34]. *L. coryniformis* CRL 1001 is the first facultative heterofermentative strain described as cobalamin producer.

**Fig. 4:**
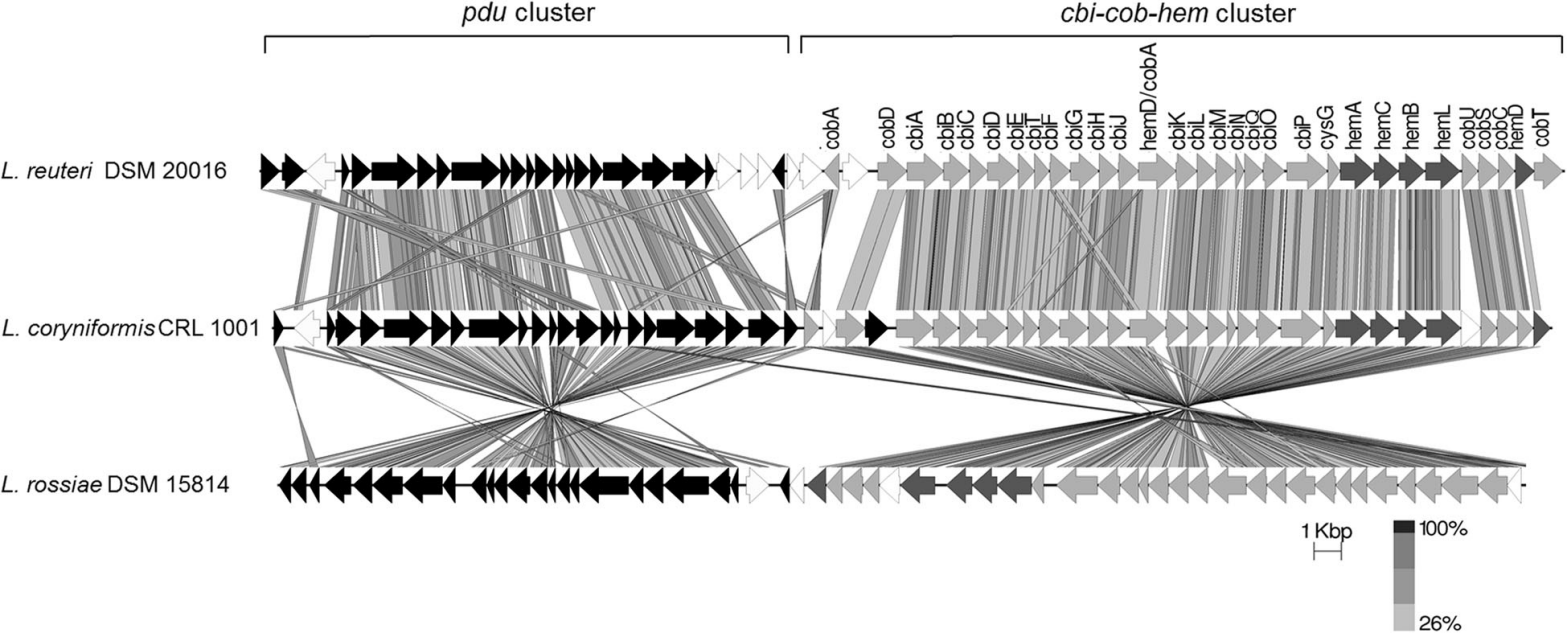
Linear comparison of the *pdu* cluster and the *cbi-cob-hem* cluster between *L. coryniformis* CRL 1001, *L. reuteri* DSM 20016 (up) and *L. rossie* DSM 15814 (down). Genes in the gene clusters are depicted by arrows indicating the transcription direction. Genes conserved between the three genomes are colored in grey and black bars indicating orthologous regions

Comparative genomic studies among *L. coryniformis* CRL 1001, *L. reuteri* DSM 20016 and *L. rossiae* DSM 15814 strains evidenced a conserved genetic organization of the coenzyme B_12_ biosynthetic genes. In these three strains, the *cbi*-*cob*-*hem* cluster is adjacent to the *pdu* cluster (Fig. 4). However, contrasting other cobalamin producer strains, this two gene sets (*pdu* and *cbi*-*cob*-*hem*) have an opposite orientation in the *L. rossiae* genome. Morita et al. [10] suggested that both gene cluster might have been acquired by horizontal transfer and inserted independently in these genomes.

As previously described for cobalamin producer anaerobic strains, the *hem* genes were located among the *cbi-cob* genes in all the organisms under comparison.

The *cblS* and *cblT* genes identified in the genome of *L. coryniformis* were also detected in the genome of *L. rossiae* where they have an identical localization in the *cbi*-*cob*-*hem* cluster. However, these genes are absent in the genome of *L. reuteri*. The *cobT* gene, encoding a Nicotinate mononucleotide (NaMN): base phosphoribosyltransferase, has only been described in this latter specie for the strains *L. reuteri* CRL 1098 (AY780645.1), *L. reuteri* JCM 1112 (NC_010609.1) and *L. reuteri* DSM 20016 (NZ_AZDD00000000.1).

Until now, among the *Lactobacillus* strains described as cobalamin producers, *L. coryniformis* CRL 1001 is the only strain harbouring the gene encoding for PduX on its genome (Fig. 4).

A comparative analysis between the deduced amino acid sequences of each ORF included in the vitamin B_12_ biosynthetic cluster of *L. coryniformis* CRL 1001 and those from *L. rossiae*, *L. reuteri*, *Listeria* sp*.* and *Salmonella* sp. using the BLASTP algorithm, was performed. We found that most of *L. coryniformis* CRL 1001 proteins have the highest percentage of identity with those identified in *L. rossiae* strain (Table 1), with the exception of CobD and PduX, which were more similar to *Listeria* proteins. This result indicates that the amino-propanol phosphate synthesis from L-threonine in *L. coryniformis* CRL 1001 is similar to that performed by *Listeria* strains, previously described by Fan et al. [26]. These results suggest an alternative vitamin B_12_ biosynthetic pathway in this genus.

**Table 1:** Comparative analysis of the deduced amino acid sequences of each ORF involved in vitamin B_12_ biosynthesis cluster of *Lactobacillus coryniformis* CRL 1001 with those present in other cobalamin producer strains

*L. coryniformis* CRL 1001		*Lactobacillus rossiae*		*Lactobacillus reuteri*		*Listeria sp.*		*Salmonella sp.*	
Protein	Length (aa)	Length (aa)	Identity (%)	Length (aa)	Identity (%)	Length (aa)	Identity (%)	Length (aa)	Identity (%)
CobA	188	189	71	194	68	188	56	176	40
Cb1 T	166	174	61	-*	-*	164	54	192	36
Cob D	360	364	41	362	42	361	45	364	42
Pdu X	288	-*	-*	-*	-*	291	37	288	36
Cbi A	456	457	59	454	61	452	54	459	50
Cbi B	312	319	62	319	58	316	55	459	50
Cbi C	217	213	68	228	64	210	64	210	51
Cbi D	365	375	74	383	73	373	55	370	50
Cbi E	199	200	54	199	55	198	53	201	43
Cbi T	184	188	56	184	63	189	53	192	44
Cbi F	254	258	83	253	77	249	76	257	71
Cbi G	351	355	55	351	53	343	46	351	36
Cbi H	241	241	77	241	78	241	67	241	58
Cbi J	249	250	47	252	56	250	45	263	33
Hem D/Cob A	449	468	45	464	48	493	40	-*	-*
Cbi K	262	259	47	259	53	261	49	264	46
Cbi L	235	232	47	232	62	236	48	237	38
Cbi M	248	239	77	247	74	244	59	245	57
Cbi N	104	114	76	103	63	82	56	93	45
Cbi Q	235	230	54	225	56	225	37	225	34
Cbi O	276	274	65	269	63	268	54	271	51
Cbi P	499	499	67	503	70	511	57	506	56
Cys G	153	148	45	152	51	146	44	154	32
Hem A	429	431	53	421	55	-	-	-	-
Hem C	305	309	55	305	55	-	-	-	-
Hem B	324	321	81	323	77	-	-	-	-
Hem L	430	430	77	431	75	-	-	-	-
Cbl S	242	252	53	-*	-*	252	38	273	37
Cob U	196	197	46	196	54	185	41	181	40
Cob S	253	257	52	253	53	248	38	244	35
Cob C	194	194	46	196	43	191	32	202	29
Hem D	209	243	46	236	49	-	-	-	-

Length variation among deduced aminoacid sequences of each ORF related to cobalamin production in *Lactobacillus*, *Salmonella* and *Listeria* strains. The percentage of protein sequence identity is related to those detected in *Lactobacillus coryniformis* CRL1001. (-*) ORF not detected. (-) ORF not detected among *cbi*-*cob*-*hem* cluster

## Conclusion

In this work, we demonstrated for the first time that *L. coryniformis* subsp. *coryniformis* CRL 1001 is able to produce corrinoids with cobalamin activity. The chemical characterization of the molecule isolated from CRL 1001 CE using HPLC and mass spectrometry show that the majority of corrinoid produced is pseudocobalamin that has adenine like Coα-ligand instead 5,6-dimethylbenzimidazole. This molecule is one of the forms of coenzyme B_12_ occurring in nature. Genomic studies revealed the presence of the complete anaerobic biosynthetic pathway of coenzyme B_12_. The comparative analysis of the cobalamin gene cluster of *L. coryniformis* CRL 1001 with those present in strains previously described as vitamin producers, revealed that this strain bears the genes *cblS*-*cblT* only present in the genome of *L. rossiae* DSM 15814. Moreover, *pduX* gene has not been detected in another strain of the *Lactobacillus* genus. The results of this work provide the first evidence of vitamin B_12_ synthesis by a *L. coryniformis* strain.

## Methods

### Strains, media and culture conditions

*L. coryniformis* CRL 1001, a strain originally isolated from silage, belongs to the CERELA-CONICET culture collection. The CRL 1001 strain was grown in Man-Rogosa-Sharpe (MRS) broth and Vitamin B_12_ Assay Medium (Merck, Germany) overnight at 37 °C without shaking. *S.* Typhimurium AR 2680, a strain with mutations in the genes *metE* and *cbiB*, was used as indicator strain in bioassays for cobalamin determination. This strain was grown in Luria-Bertani (LB) broth at 37 °C with aeration. To perform the bioassay, the indicator strain was grown in minimal A medium (NaCl, 0.5 g L^−1^, Na_2_HPO_4_, 6 g L^−1^; KH_2_PO_4_, 3 g L^−1^, NH_4_Cl 1 g L^−1^; glucose, 4 g L^−1^; MgSO_4_, 2 mM, CaCl_2_ 0.1 mM). As negative control, *L. plantarum* ATCC 8014 was employed.

### Preparation of cultures and cell-extracts

The production of corrinoid type-cobalamin by *L. coryniformis* CRL 1001 was detected as following: a culture was inoculated duplicated in vitamin B_12_-free assay medium, grown at 37 °C for 16 h and subcultured three times in the same medium before use. Cell Extracts (CE) were prepared from 100 mL of culture grown for 24 h; after harvesting (8000 × *g*, 5 min), cells were washed in 10 mL of 0.1 M phosphate buffer pH 7.0 twice and resuspended in 10 mL of the same buffer and were fasted overnight at 2 °C. Cells were harvested (8000 × *g*, 5 min) and resuspended in 10 mL of extraction buffer (0.1 M Na_2_HPO_4_, pH 4.5 reached with, 0.005 % KCN). The cell suspension obtained was autoclaved at 120 °C for 20 min, the pellet was separated by centrifugation (10,000 × *g*, 20 min), and the supernatant was passed over a solid-phase extraction (SPE) column C18-E (Phenomenex) with 500 mg particle size and 6 mL reservoir volume; the column was previously activated with 6 mL of methanol HPLC degree (Loba Chemie, India). The column was washed with 2 volumes of ultra-purified water twice to remove salts and other hydrophilic contaminants. The potentials corrinoid was eluted with 1 volume of methanol and concentrated to dryness in vacuum at 30 °C. The residue was dissolved in 100 μl of 50 % methanol and stored in the dark at - 20 °C until use.

### Cobalamin detection

To demonstrate the production of cobalamin by *L. coryniformis* CRL 1001, a bioassay using *S.* Typhimurium AR 2680 as indicator strain was performed. The CE from *L. coryniformis* CRL 1001 was examined for its ability to correct the cobalamin requirement of the AR 2680 strain in minimal medium. For this purpose, *S.* Typhimurium AR 2680 cells were grown for 16 to 18 h in LB medium; the active culture was seeded onto minimal agar medium specific for this strain. Four wells were performed in each agar plate. Fifteen microliters of CE from *L. coryniformis* CRL 1001 (dilutions 1 10^−1^ and 1 100^−1^) were loaded in each well and incubated 24 h under anaerobic conditions at 37 °C. As comparative standard of vitamin B_12_, a commercial cianocobalamin (CN-Cbl) solution (0.5 μg mL^−1^) (Sigma-Aldrich, cod. V2876) was used. The CE of *L. plantarum* ATCC 8014, obtained in a similar way that the CE of *L. coryniformis* CRL 1001, was used as negative control.

### Isolation and characterization of the corrinoid

To isolate the corrinoid produced by *L. coryniformis* CRL 1001, the CE was loaded onto a C_18_ solid-phase 5 μm extraction column (250 × 4.6 mm) (Shimadzu Shim-Pack VP-ODS) and purified by reverse-phase (RP)-HPLC using a LC_20 AT system automated gradient controller with a SIL-20ª HT autosampler and a SPD-M20A diode array UV–visible detector at 361 nm (Shimadzu Corporation, Kyoto, Japan). The column was thermostated at 25 °C. The mobile phases was filtered through a 0.22 μm membrane filter and vacuum degassed prior to use. The mobile phases were prepared with ultra-purified water with 0.1 % formic acid (Solvent A) and methanol with 0.1 % (v/v) formic acid (Solvent B).

The protocol used was described by Yu et al. [35] with some modifications. The following mobile phase gradients (Solvent B) were used: 0–2 min (20 %); 2–5 min (35 %); 5–22 min (100 %); 22–26 min (100 %); 26–36 min (20 %); and 36–45 min (5 %); this step was followed by the final passage of Solvent B at 5 % for re-optimization of the parameters in the module HPLC. The volume of injection was 100 μl. The HPLC was operated in a constant flow mode and the flow rate was kept at 0.5 ml min^−1^.

The peaks with RT close to the RT of CN-Cbl standard (Sigma-Aldrich, cod. V2876) (concentration = 5 μg mL^−1^) were collected and concentrated to dryness under vacuum at 30 °C. Then, the peaks were dissolved with methanol 50 % and stored in the dark at - 20 °C until use. The vitamin B_12_ activity of each collected peak at 1.10^−1^ and 1.100^−1^ dilutions was detected using the bioassay with the *Salmonella* strain. Peaks with vitamin B_12_ activity positive were analysed by mass spectrometry.

The active peaks collected from the RP-HPLC were analysed using Ultimate 3000 RSLC Dionex - Thermo Scientic with a C_18_ column 50 × 2.1 mm that contained 1.9 micron particles (Hypersil-GOLD). The solvents for Liquid Chromatography were solvent A (ultra-purified water with 0.1 % formic acid) and solvent B (methanol with 0.1 % formic acid). The flow rate was 0.2 mL min^−1^ and the injection volume was 10 μL. Chromatography was in gradient, and the solvent composition was chosen such that the compound of interest eluted 8–9 min after injection. The mobile phase gradients (solvent B) were maintained during a 35 min run: 0–2 min (5 %); 2–5 min (35 %); 5–22 min (100 %); 22–24 min (100 %); 24–28 min (5 %); and 28–35 min (5 %), this step was followed by 50 % of solvent B for re-optimization of the parameters in the module HPLC coupled with Mass. A single peak for each sample was eluted and the area of the signal was used for quantitative analysis. Positive-ion MS/MS experiments were performed in product mode on a triple quadrupole mass spectrometer (Thermo Scientific TSQ Quantum Access Max).

### Genome sequencing

The genomic DNA was extracted from the cultured bacterium according to *Pospiech and Neuman* [36] and the genome sequence was determined using a whole-genome shotgun (WGS) strategy with an Ion Torrent personal genome machine (Life Technologies). Quality filtered reads were *in silico* assembled via the DNASTAR NGen assembler by MR DNA (Shallowater, TX).

This Whole Genome Shotgun project has been deposited at DDBJ/EMBL/GenBank under the accession LNUL00000000. The version described in this paper is LNUL01000000.

### Genomic analysis

The *L. coryniformis* CRL 1001 genome sequence analysis was carried out following different approaches. Open Reading Frame (ORF) prediction and functional assignment was automatically performed using the RAST webserver (http://rast.nmpdr.org/), ORF Finder (http://www.ncbi.nlm.nih.gov/gorf/gorf.html) and BLASTX (http://blast.ncbi.nlm.nih.gov/Blast.cgi). The classification of the ORFs was carried out using BLASTP alignment against the database (http://blast.ncbi.nlm.nih.gov/Blast.cgi), using a cut-off E-value of 1.10^−4^ with at least 30 % of identity across a minimum of 50 % of a given protein length.

### Comparative analyses

BLAST-based comparative analyses were performed of each one of the CDS of the *cbi-cob-hem* cluster between *L. coryniformis* CRL 1001 and two well-characterized *Lactobacillus* strains described as corrinoids compound producers with cobalamin activity, *L. reuteri* DSM 20016 and *L. rossiae* DSM 15814. This analysis was performed employing Easyfig 2.1: a genome comparison visualizer application [37].
